# Beneficial Effects of Vitamins K and D3 on Redox Balance of Human Osteoblasts Cultured with Hydroxyapatite-Based Biomaterials

**DOI:** 10.3390/cells8040325

**Published:** 2019-04-08

**Authors:** Ewa Ambrożewicz, Marta Muszyńska, Grażyna Tokajuk, Grzegorz Grynkiewicz, Neven Žarković, Elżbieta Skrzydlewska

**Affiliations:** 1 Department of Analytical Chemistry, Medical University of Bialystok, 15-222 Bialystok, Poland; ewa.ambrozewicz@umb.edu.pl (E.A.); marta.muszynska@umb.edu.pl (M.M.); 2 Department of Integrated Dentistry, Medical University of Bialystok, 15-230 Bialystok, Poland; grazyna.t1@gmail.com; 3 Pharmaceutical Research Institute, 01-793 Warsaw, Poland; g.grynkiewicz@ifarm.eu; 4 Laboratory for Oxidative Stress, Rudjer Boskovic Institute, 10000 Zagreb, Croatia; zarkovic@irb.hr

**Keywords:** hydroxyapatite-based biomaterials, osteoblast growth, redox balance, vitamins, lipid peroxidation, 4-hydroxynonenal, oxidative stress

## Abstract

Hydroxyapatite-based biomaterials are commonly used in surgery to repair bone damage. However, the introduction of biomaterials into the body can cause metabolic alterations, including redox imbalance. Because vitamins D3 and K (K1, MK-4, MK-7) have pronounced osteoinductive, anti-inflammatory, and antioxidant properties, it is suggested that they may reduce the adverse effects of biomaterials. The aim of this study was to investigate the effects of vitamins D3 and K, used alone and in combination, on the redox metabolism of human osteoblasts (hFOB 1.19 cell line) cultured in the presence of hydroxyapatite-based biomaterials (Maxgraft, Cerabone, Apatos, and Gen-Os). Culturing of the osteoblasts in the presence of hydroxyapatite-based biomaterials resulted in oxidative stress manifested by increased production of reactive oxygen species and decrease of glutathione level and glutathione peroxidase activity. Such redox imbalance leads to lipid peroxidation manifested by an increase of 4-hydroxynonenal level, which is known to influence the growth of bone cells. Vitamins D3 and K were shown to help maintain redox balance and prevent lipid peroxidation in osteoblasts cultured with hydroxyapatite-based biomaterials. The strongest effect was observed for the combination of vitamin D3 and MK-7. Moreover, vitamins promoted growth of the osteoblasts, manifested by increased DNA biosynthesis. Therefore, it is suggested that the use of vitamins D3 and K may protect redox balance and support the growth of osteoblasts affected by hydroxyapatite-based biomaterials.

## 1. Introduction

Hydroxyapatite-based biomaterials possess osteoconductive properties and can also act as three-dimensional scaffolds to support bone regeneration [1]. However, introducing biomaterials into the body can lead to metabolic alterations. Namely, interactions between osteoblasts and biomaterials may interfere with cellular metabolism, including redox balance leading to oxidative stress [2]. The ingredients released by biomaterials may also take part in this activity [3,4]. Compounds released from bone substitutes can have adverse effects on the viability and function of osteoblasts, even in the absence of physical contact [2,5,6]. There is also evidence that the production of pro-inflammatory cytokines by osteoblasts is increased in the presence of hydroxyapatite [7]. The lack of cytocompatibility of bone substitutes can hinder bone regeneration and prolong wound healing [8]. To reduce the cytotoxicity of biomaterials and enhance their desirable bioactivities, they are often modified by the inclusion of various compounds, including growth factors [9] and antioxidants, e.g., N-acetylcysteine [5], or combined with other components, such as matrix-derived proteins [10]. 

The physiology of bone metabolism is dependent on the synergistic interplay between vitamins D3 and K [11]. The active form of vitamin D3 is 1,25-dihydroxycholecalciferol (1,25(OH)2D3; calcitriol). Calcitriol forms a complex with a specific nuclear receptor, commonly known as the vitamin D receptor (VDR), to control the expression of key bone-related proteins in osteoblasts, including osteocalcin (OC), and to regulate calcium and phosphate homeostasis [12,13]. The antioxidant and anti-inflammatory properties of vitamin D3 have been confirmed both in vitro and in vivo. Under pathological conditions, vitamin D3 has been shown to enhance the level and activity of antioxidant proteins which protect cellular proteins from oxidative modification. Vitamin D3 activates the Nrf2-Keap1 antioxidant pathway leading to transcription of antioxidant proteins and has been shown to reduce levels of advanced glycation end-products in the aortic wall of diabetic rats [14,15]. Administering vitamin D3 to rats increased the activity of an antioxidant enzyme superoxide dismutase (SOD) in the liver. Furthermore, a decrease in vitamin D3 levels in the blood serum in obese children was correlated with a decrease in SOD levels [16,17,18,19]. Changes in antioxidant capacity correspond to an increase in lipid peroxidation [20]. Vitamin D3 also inhibits oxidative stress and endoplasmic reticulum stress in endothelial cells thereby reducing the risk of cardiovascular disease in humans [21] and preventing endothelial cell death [22]. In addition, vitamin D3 plays a role in T cell-mediated immunity [23].

The vitamin K family is a group of fat-soluble vitamins that regulate metabolic processes [24]. The two main forms of vitamin K are phylloquinone (K1) and menaquinone (K2), including the menaquinone-4 (MK-4) and menaquinone-7 (MK-7) homologues. Vitamin K1 is transported to the liver where it regulates the production of coagulation factors, whereas vitamin K2 is found in many tissues, including bone, where it regulates the activity of vitamin K-dependent proteins, such as matrix carboxyglutamic acid (Gla)-protein (MGP) and OC (GLA protein) [25]. Bone metabolism depends on interaction between vitamins D3 and K2. Vitamin D promotes vitamin K-dependent protein production while vitamin K activates proteins involved in bone metabolism. Vitamin K acts as a cofactor in the carboxylation of glutamic acid (Glu) to Gla and the metabolically active form of OC, which can bind and deposit calcium in the extracellular matrix [11,26]. In vitro, vitamin K2 was shown to promote osteoclast apoptosis in a dose-dependent manner and to suppress osteoblast apoptosis [27,28]. Moreover, it was found that vitamin K2 increases the number and activity of osteoclasts [28] and the MK-4 homologue promotes osteoblast differentiation and proliferation of osteoclasts [29].

Vitamin K participates in blood coagulation, signal transduction processes, and cell proliferation [30], and has the ability to alter redox balance in cells [31]. In its reduced form, vitamin K hydroquinone (KH2) protects phospholipid membranes from peroxidation by direct reactive oxygen species (ROS) uptake [32]. During the vitamin K cycle, hydroquinone is reconstituted with the participation of *vitamin K epoxide reductase complex subunit 1* (VKORC1) [31]. Vitamin K inhibits the activation of 12-lipoxygenase (12-LOX) to prevent the formation of ROS [33]. The antioxidant actions of vitamins K1 and K2 (MK-4) protect oligodendrocytes and neurons from oxidative stress caused by glutathione (GSH) deficiency [34]. Nanomolar concentrations of vitamins K1 and K2 (MK-4) were also shown to prevent oxidative stress-induced neuronal death [33]. Since oxidative stress is directly associated with inflammation, vitamin K may reduce oxidative stress by lowering levels of pro-inflammatory factors [35,36]. 

There is a growing body of evidence to suggest vitamin K and vitamin D have synergistic effects on bone metabolism [11,37,38]. The aim of the present study was to investigate the effect of vitamin D3 and K on cellular metabolism and to determine whether vitamins D3, K1, MK-4, and MK-7 could be potentially used to support bone regeneration alongside hydroxyapatite-based biomaterials. Therefore, redox balance in osteoblasts cultured in presence of biomaterials was determined. The biomaterials characterized by origin—human (Maxgraft), bovine (Cerabone), porcine (Apatos and Gen-Os), and composition—hydroxyapatite (Maxgraft, Cerabone, Apatos) and hydroxyapatite enriched with collagen (Gen-Os) were examined. 

## 2. Materials and Methods

### 2.1. Materials

To evaluate the in vitro effect of vitamin K (K1, MK4, and MK7) and vitamin D3 on osteoblasts’ metabolism cultured with biomaterial cells were cultured on commercial biomaterials of different origin and chemical composition. Vitamin K1 (Sigma–Aldrich, St. Louis, MO, USA), vitamin K2 (Menaquinone 4, MK4 (Sigma–Aldrich, St. Louis, MO, USA) and Menaquinone 7, MK7 (was chemically synthesized by the Pharmaceutical Research Institute, Warsaw, Poland)), and vitamin D3 (1,25-dihydroxyvitamin D3 (1,25(OH)2D3) (Santa Cruz Biotechnology, USA)), was added to cells. Four different hydroxyapatites: human origin (Maxgraft, Botiss biomaterials GmgH, Germany), bovine origin (Cerabone, Botiss Biomaterials GmbH, Germany), porcine origin (Apatos, Tecnoss, Italy), and porcine hydroxyapatite with preserved collagen (Gen-Os, Tecnoss, Italy) were used for experiments.

### 2.2. Cell Cultures and Treatments Used

All experiments were performed using a human fetal osteoblast cell line (hFOB 1.19) obtained from ATCC (American Type Culture Collection, Menassas, VA, USA). The optimal time to assess metabolic changes during osteoblast differentiation is about 20 days [39]. During this period, three stages of osteoblast differentiation can be distinguished: proliferation (until day 4), synthesis of extracellular matrix (between 12–16 days), and mineralization of the extracellular matrix of the bone (about day 20) [39,40]. Cells were cultured for 20 days at 39 °C in 5% CO_2_ in air in a 1:1 mixture of Ham’s F12 medium and Dulbecco’s modified Eagles’s medium containing 2.5 mmol/L l-Glutamine without phenol red and supplemented with 0.3 mg/mL G418, 100 U/mL penicillin, 100 μg/mL streptomycin, and 10% fetal bovine serum (FBS). Culture medium was renewed every 2 days. 

For all experiments, osteoblasts were seeded on each of the hydroxyapatite-based biomaterials (Maxgraft, Cerabone, Apatos, Gen-Os; 100 mg/well) at a density of 1 × 10^5^ cells/well in 6-well plates. The influence of vitamin D3 and vitamin K on osteoblast growth was investigated by dividing the cells into several treatment groups: cells treated with vitamin D3, cells treated with vitamin K (K1, MK-4, or MK-7), and cells treated with combinations of vitamin D3 and K (D3+K1, D3+MK-4, or D3+MK-7). Controls without any hydroxyapatite were cultured in parallel. Cells were supplemented with vitamin K1 and K2 (MK-4 and MK-7) at a concentration of 10 µmol/L [29,41] and/or vitamin D3 at concentration 1 nM in the presence of 10 mmol/L sodium β-glycerophosphate to accelerate the mineralization process. After 4, 8, 12, 16, and 20 days, cells were collected. Vitamin D3 was used in its metabolically active form of 1,25(OH)2D3, which has previously been shown to regulate osteoblast differentiation [42,43]. The MTT (*3-(4,5-dimethylthiazol-2-yl)-2,5-diphenyltetrazolium bromide)* colorimetric assay was used to study the effect of vitamin D3 on cells and to determine the optimal concentration of vitamin D following a procedure previously described in the literature [44]. The concentration of vitamin K used for the MTT assay did not affect cell viability.

To examine the effects of vitamins D3, K1, K2 (MK-4 and MK-7), and hydroxyapatite on osteoblast proliferation and differentiation, DNA content as well as alkaline phosphatase (ALP) and OC levels were determined. Redox status was estimated for each group based on measured ROS, GSH, and 4-hydroxynonenal (4-HNE) levels. 

### 2.3. Determination of ROS Level

Electron spin resonance (ESR) spectrometer e-scans (Noxygen GmbH/Bruker Biospin GmbH, Germany) were used to detect total ROS generation using spin probe CMH (1-hydroxy-3-methoxycarbonyl-2,2,5,5-tetra-methylpyrrolidine, 200 μmol/L), which selectively interacts with ROS to form a stable nitroxide CM-radical (carbamoyl radical) with a half-life of 4 h [43]. After 4, 8, 12, 16, and 20 days in culture, the cells were suspended in Krebs HEPES buffer (2-[4-(2-hydroxyethyl)piperazin-1-yl]ethanesulfonic acid) (KHB) and the CMH spin probe was added. Samples were incubated for 30 min at 37 °C in a mixture of N2 and 02 of 96:4 (02:N2) and electron paramagnetic resonance (EPR) was performed on cell lysates using the following acquisition parameters: field center 1.99 g, microwave power 20 mW, amplitude of modulation 2 G, sweep time 10 s, number of scans 10, range of deviation 60 G.

### 2.4. Determination of GSH Level

Non-enzymatic antioxidant—glutathione (GSH)—was quantified using capillary electrophoresis (CE) method of Maeso et al. [45]. Cells were sonicated with a mixture containing acetonitrile/water (ACN/H2O 62.5:37.5, *v*/*v*) and centrifuged at 30,000× *g* for 10 min. The separation of supernatant components was performed on a capillary with 47 cm total length (40 cm effective length) and 50 μm i.d. and was operated at 27 kV with UV detection at 200 ± 10 nm.

### 2.5. Determination of Glutathione Peroxidase Activity

Glutathione peroxidase (GSH-Px, EC 1.11.1.6) activity was estimated by spectrophotometry using a microplate reader (Infinite M200, Tecan), according to the method of Paglia and Valentine [46]. Enzyme activity was measured using an indirect method involving two conjugated reactions: glutathione oxidation reaction (catalyzed by glutathione peroxidase) and glutathione reduction reaction with simultaneous oxidation of reduced nicotinamide adenine dinucleotide phosphate (NADPH) to NADP. The cell lysate was combined with a mixture containing 1.5 mM ethylenediaminetetraacetic acid (EDTA; in 0.2 mol/L Tris-HCl buffer; pH 7.6), 15 mmol/L sodium azide, and 0.72 mmol/L NADPH in a 96-well plate and incubated for 5 min at 20 °C. Then glutathione reductase and 0.1 ml of 10 mmol/L H_2_O_2_ in 0.02 mmol/L Tris-HCl buffer (pH 7.6) were added to each sample. Absorbance was measured at 340 nm for 1 min relative to Tris-HCl buffer. One unit of GSH-Px activity was defined as the amount of enzyme catalyzing the oxidation of 1 µmol NADPH min^−1^ at 25 °C and pH 7.4. Enzyme specific activity was expressed as micro-units per mg of protein.

### 2.6. Determination of Lipid Peroxidation

Lipid peroxidation was estimated based on the level of 4-hydroxynonenal (4-HNE) as a derivative of O-pentafluorobenzyl-oxime-trimethyl silyl ether (O-PFB-oxime-TMS) [47], assessed using gas chromatography-mass spectrometry (GC/MS) in selected ion monitoring mode (SIM), according to a previously described method [48]. Briefly, aldehydes were derivatized by the addition of O-(2,3,4,5,6-pentafluorobenzyl) hydroxylamine (PFB) in PIPES buffer (piperazine-N,N′-bis(2-ethanesulfonic acid) in the presence of benzaldehyde-D6 as an internal standard (IS). The O-PFB-oxime aldehyde derivative was extracted using hexane. The hexane phase was evaporated under a stream of argon followed by the addition of N,O-bis(trimethylsilyl)trifluoroacetamide in 1% trimethylchlorosilane to form TMS ether of the hydroxyaldehyde group. A 1 µl aliquot was injected into the GC-MS. Derivatized aldehyde was analyzed using a 7000 quadrupole MS/MS (7890A GC, Agilent Technologies, USA) equipped with a HP-5 ms capillary column (0.25-mm internal diameter, 0.25-µm film thickness, 30-m length). The column temperature was initially set to 50 °C for 1 min, increased at a rate of 10 °C/min to 200 °C, then at 3 °C/min to 220 °C, 20 °C/min to 310 °C, and finally, maintained at 310 °C for 5 min. The injector temperature was maintained at 250 °C, the transfer line was held constant at 280 °C, and the source temperature was set to 230 °C. Derivatized aldehyde was detected by selected ion-monitoring GC/MS using the following ions: *m*/*z* 333.0 and 181.0 for 4-HNE-PFB-TMS and *m*/*z* 307.0 for nternal standard (IS) derivative.

### 2.7. DNA Proliferation Assay 

A DNA fluorometric assay for measuring osteoblasts proliferation was used [49]. Briefly, medium was discarded, and cells were washed with a washing solution (0.8% NaCl, 0.04% KCl, 0.1% ethylenediaminetetraacetic acid EDTA, and 1% NaN3). The plates were then allowed to dry at room temperature for 10 min, wrapped with parafilm, and frozen at −70 ° C until analysis. After removal from the freezer, 0.01% dodecyl sodium sulfate (SDS) was added, and samples were incubated at room temperature for 15 min. Cell lysates in the amount of 10 μL were put into a 96-well plate with H33258 at a concentration of 2 µg/mL. The DNA level was measured under excitation of 360 nm and emission at 460 nm and calculated from the calibration curve for Calf Thymus DNA solution. The DNA level was expressed as ng of DNA per mg of protein.

### 2.8. Determination of ALP Activity

Alkaline phosphatase (ALP) activity was determined fluorometrically using the p-nitrophenyl phosphate (p-NPP) according to Reference [50] at 405 nm. Briefly, a lysis buffer (10 mmol/L Tris-HCl, pH 7.5, containing 0.5 mmol/L MgCl2, 0.1% Triton X-100) was added to cells, and after 10 min incubation on ice, cells were collected in tubes and stored at −70 °C until analysis. After thawing, samples were sonicated and centrifuged at 10,000× *g* for 15 min at 4 °C. Next, glycine buffer (25 mmol/L, pH 10.4) containing 2 mmol/L MgCl2 and 10 mmol/L p-NPP was added to samples, and then incubated at 37 °C for 25 min. The enzymatic reaction was stopped by the addition of 3 mol/L NaOH. The activity of ALP was expressed as the amount of enzyme (nmol) that catalyzed the reaction with substrate per minute per mg of protein.

### 2.9. Determination of OC Level

The osteocalcin (Gla-OC) concentration was determined in the culture medium. The osteocalcin level was determined using a commercially available human-specific enzyme-linked immunosorbent assay (ELISA) kit (Gla-type Osteocalcin (Gla-OC) EIA Kit, TaKaRa, Bio Inc.). The tests were performed according to the manufacturer’s protocol. The OC level was expressed as ng of osteocalcin per ml of culture media.

### 2.10. Statistical Analysis

Data were analyzed using standard statistical analyses, one-way/two-way analysis of variance (ANOVA) to determine significant differences between different groups. All analyses on cells were performed on the results obtained from three independent experiments. The results are expressed as the mean ± standard deviation (SD) for *n* = 3. *p*-values less than 0.05 were considered significant. 

## 3. Results

### 3.1. Biochemical Studies

**Figure 1:**
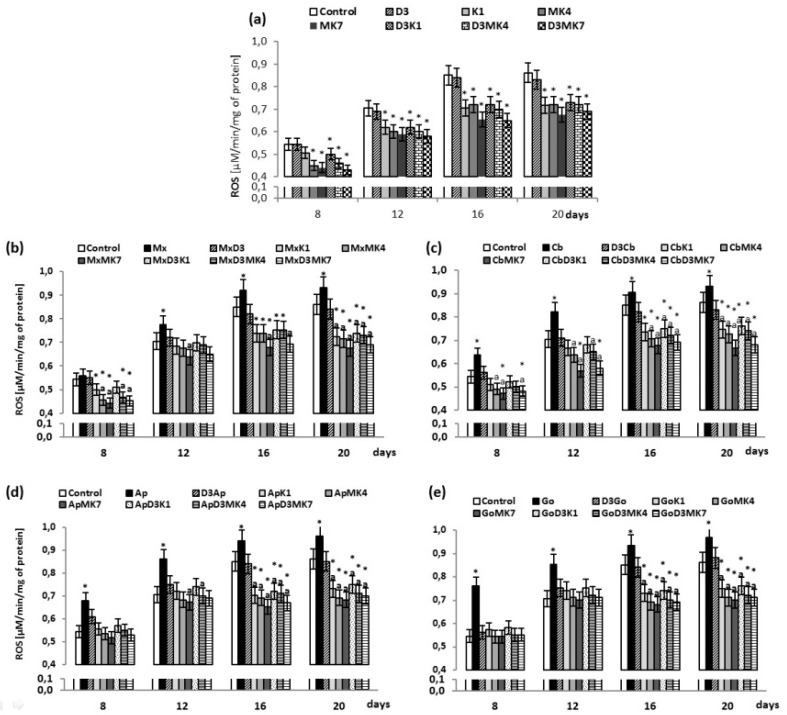
Reactive oxygen species (ROS) level in osteoblasts after incubation with hydroxyapatites and treated with vitamins D3, K1, MK4, and MK7 after 8, 12, 16, and 20 days. The results are expressed as the μM/min/mg of protein and are shown as the mean ± SD (*n* = 5). The values for the control cells and the treated cells were significantly different according to unpaired Student’s *t*-test. * Statistically significant differences versus control, *p* < 0.05; (**a**) statistically significant differences versus group hydroxyapatites (Mx (Maxgraft), Cb (Cerabone), Ap (Apatos), Go (Gen-Os), respectively for graphs (**b**–**e**)); p < 0.05.

The results obtained show that the growth of osteoblasts was associated with a gradual increase of ROS levels, the largest increase being observed between the days 8 and 12 (Figure 1). While vitamin D3 did not affect such changes in ROS levels for cultured osteoblasts, the other vitamins attenuated continuous increases of ROS. The most prominent reduction of ROS production was observed in MK-7-treated osteoblasts at day 12 and day 16 (16% and 23%, respectively), whereas ROS levels observed in the K1-treated osteoblasts decreased by 11–17% and in MK-4-treated osteoblasts, by 14–15%. 

Opposite to the vitamins, the hydroxyapatite-based biomaterials further enhanced production of ROS by cultured osteoblasts, which was also shown to be significantly reduced by vitamins, depending on the type of biomaterials and vitamins used (Figure 1b–e). Osteoblasts cultured on hydroxyapatite in medium containing vitamin D3 with either vitamin K1 or K2 exhibited significantly lower ROS production, particularly at day 16 and 20, compared to controls (10–20%) or cells grown on hydroxyapatite-based biomaterials alone (10–20%). 

**Figure 2:**
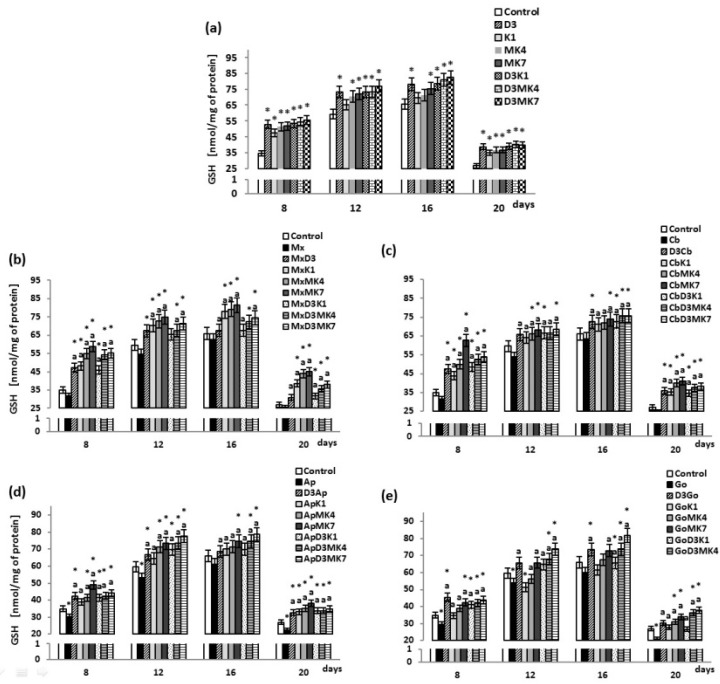
Glutathione (GSH) level in osteoblasts after incubation with hydroxyapatites and treated with vitamins D3, K1, MK4, and MK7 after 8, 12, 16, and 20 days. The results are expressed as the nmol/mg of protein and are shown as the mean ± SD (*n* = 5). The values for the control cells and the treated cells were significantly different according to unpaired Student’s *t*-test. * Statistically significant differences versus control, *p* < 0.05; (**a**) statistically significant differences versus group hydroxyapatites (Mx (Maxgraft), Cb (Cerabone), Ap (Apatos), Go (Gen-Os), respectively for graphs (**b**–**e**)); p < 0.05.

Levels of the main cytosolic antioxidant, GSH, gradually increased in osteoblast cultures until day 12; however, a decrease was observed at day 20 (Figure 2). The addition of vitamin D3 as well as K1 and K2 to culture medium significantly increased GSH concentrations compared to controls. Vitamin K1 and K2 had slightly less of an effect than vitamin D3. The largest increases in GSH concentration were observed between day 8 and 20. Furthermore, the combination of vitamin D3 and vitamin K1 or K2 resulted in enhanced GSH levels compared to cultures treated with only the vitamin K variants. Cells cultured with hydroxyapatite (Apatos and Gen-Os), exhibited lower GSH levels (Figure 2b–e); moreover, the addition of vitamin D3 and a mixture of vitamins D3 and K1 or K2 led to a significant increase in GSH levels compared to controls. The largest increase was observed between day 8 and day 20 in osteoblast cultures treated with vitamin D3 and K1 or K2 in the presence of Maxgraft (17–58%) or Cerabone (28–54%).

**Figure 3:**
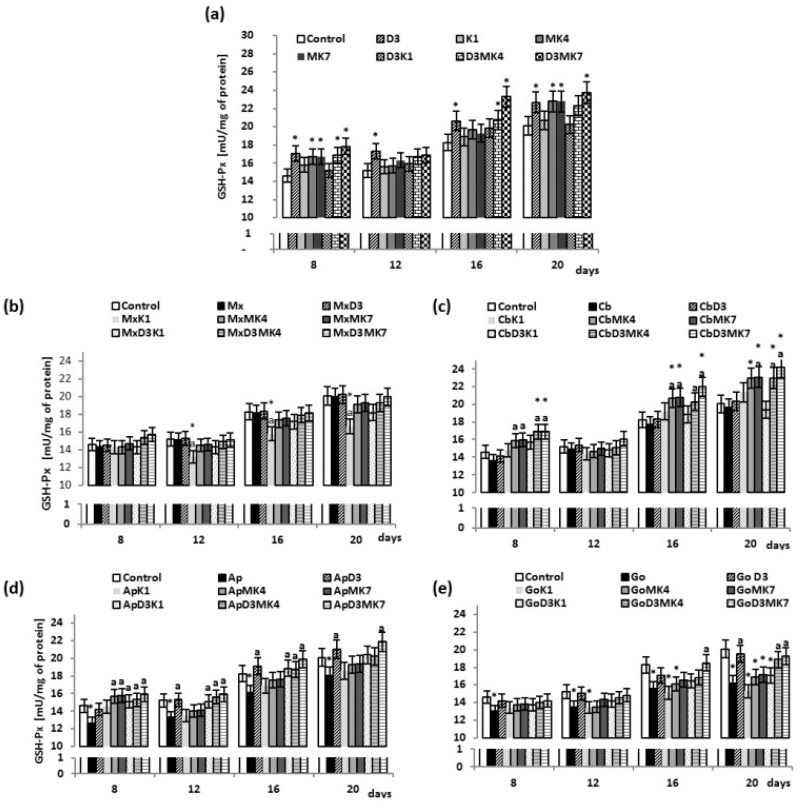
GSH-Px activity in osteoblasts after incubation with hydroxyapatites and treated with vitamins D3, K1, MK4, and MK7 after 8, 12, 16, and 20 days. The results are expressed as the mU/mg of protein and are shown as the mean ± SD (*n* = 5). The values for the control cells and the treated cells were significantly different according to unpaired Student’s *t*-test. * Statistically significant differences versus control, *p* < 0.05; (**a**) statistically significant differences versus group hydroxyapatites (Mx (Maxgraft), Cb (Cerabone), Ap (Apatos), Go (Gen-Os), respectively for graphs (**b**–**e**)); p < 0.05.

Observed changes in GSH level were associated with changes in GSH-Px activity since GSH is its co-substrate. Vitamin D3 as well as MK-4 and MK-7 significantly enhanced the activity of GSH-Px by approximately 16–13% for vitamin D3 alone between 8 and 20 days and by approximately 14% for D3+MK-4 and D3+MK-7 on day 8 and day 20, compared to controls (Figure 3a). Therefore, the combination of D3+MK-4 or D3+MK-7 significantly enhanced GSH-Px activity. The most effective treatment was D3+MK-7 which resulted in a 28% increase after 16 days. The presence of hydroxyapatite-based biomaterials, in particular Apatos and Gen-Os, led to a decrease in GSH-Px activity of approximately 11% (Figure 3b–e). Osteoblasts cultured in the presence of hydroxyapatite but treated with vitamin D3 were characterized by significantly higher GSH-Px activity (9–12% and 11–19% for Apatos and Gen-Os, respectively) compared to cells grown only in the presence of hydroxyapatite. However, a combination of vitamins, D3+MK-4 or D3+MK-7, led to a significant increase in GSH-Px activity even in the presence of hydroxyapatite. For Apatos, GSH-Px activity was enhanced by approximately 11–17% and 20% with D3+MK-4 and D3+MK-7 treatment, respectively; for Cerabone, approximately 15–24% for D3+MK-4 and 18–24% for D3+MK-7, and for Gen-Os, 7–16% for D3+MK-4, and 9–19% for D3+MK-7.

**Figure 4:**
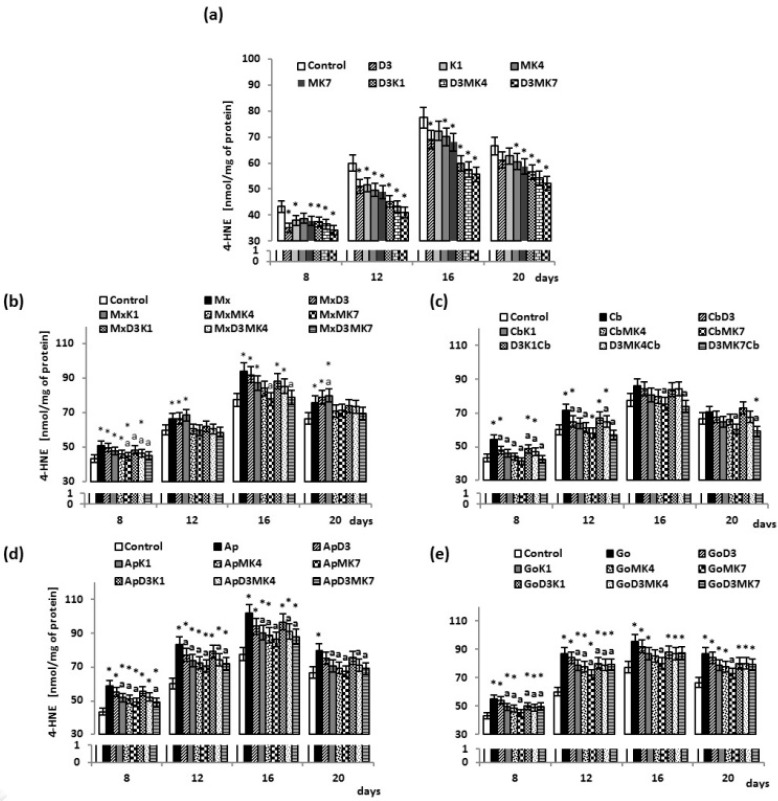
4-HNE level in osteoblasts after incubation with hydroxyapatites and treated with vitamins D3, K1, MK4, and MK7 after 8, 12, 16, and 20 days. The results are expressed as the nmol/mg of protein and are shown as the mean ± SD (*n* = 5). The values for the control cells and the treated cells were significantly different according to unpaired Student’s *t*-test. * Statistically significant differences versus control. *p* < 0.05; (**a**) statistically significant differences versus group hydroxyapatites ((Mx (Maxgraft), Cb (Cerabone), Ap (Apatos), Go (Gen-Os), respectively for graphs (**b**–**e**)), *p* < 0.05.

Furthermore, biomaterials and vitamins affected redox balance in cultured osteoblasts by modifying lipid metabolism, in the manner similar as observed for ROS levels. Namely, the growth of osteoblasts was associated with continuous increase of 4-HNE levels (Figure 4). However, different from the lack of influence of vitamin D3 on the ROS change, the most significant effects on the 4-HNE production were observed in vitamin D3-treated cultures (Figure 4a), followed by MK-7, with a reduction of approximately 12–19%. As was noticed in the case of the change of ROS (Figure 1), the presence of hydroxyapatite biomaterials also caused a significant increase in lipid peroxidation, i.e., enhanced production of 4-HNE, while vitamins attenuated lipid peroxidation induced by the osteoblast growth also if cultured in the presence of hydroxyapatite. 

### 3.2. Osteoblast Growth

**Figure 5:**
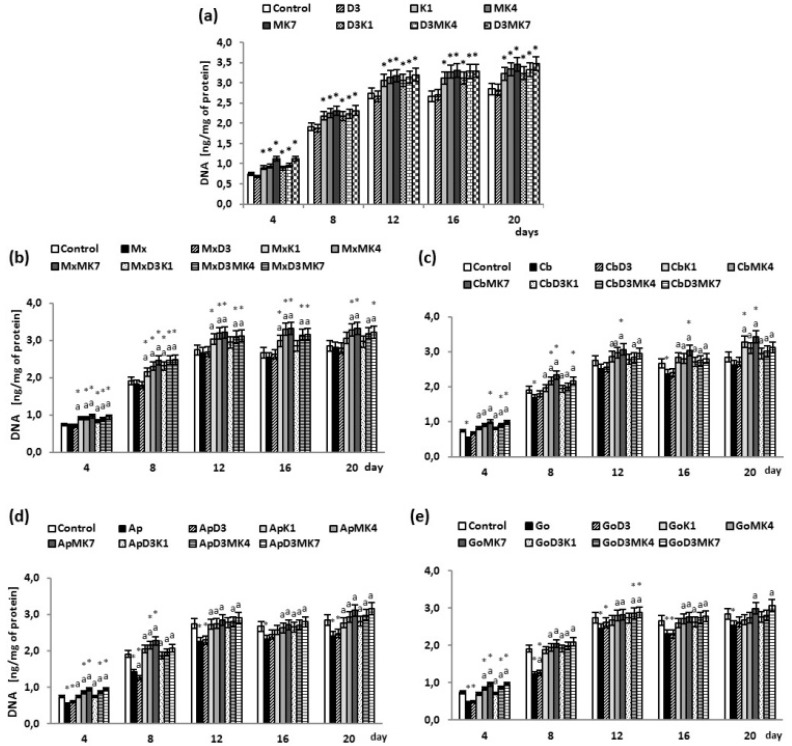
DNA levels in osteoblasts after incubation with hydroxyapatites and treated with vitamins D3, K1, MK4, and MK7 after 4, 8, 12, 16, and 20 days. The results are expressed as the ng/mg of protein and are shown as the mean ± SD (*n* = 5). The values for the control cells and the treated cells were significantly different according to unpaired Student’s *t*-test. * Statistically significant differences versus control, *p* < 0.05; (**a**) statistically significant differences versus hydroxyapatites (Mx (Maxgraft), Cb (Cerabone), Ap (Apatos), Go (Gen-Os), respectively for graphs (**b**–**e**)), *p* < 0.05;

Significant increase in the osteoblasts’ DNA levels were observed on day 12 and thereafter, remaining constant until day 20 (Figure 5). Vitamin D3 did not affect DNA levels, whereas vitamin K1 accelerated cell proliferation by 12–23%, MK-4 by 15–27%, and MK-7 by 16–51% (Figure 5a). Addition of vitamin D3 to vitamin K1 and K2 did not change the rate of osteoblast proliferation. All hydroxyapatites, with the exception of Maxgraft, inhibited osteoblast proliferation (Figure 5b–e). The most notable changes were observed for Cerabone, Apatos, and Gen-Os on day 4 and 8 of culture, with a decline in DNA levels of 23%, 24%, and 37% (day 4), and 12%, 15%, and 36% (day 8), compared to controls. When vitamin D3 was present, osteoblast proliferation was not affected. In contrast, the combinations D3+K1, D3+MK-4, and D3+MK-7 increased DNA levels in both controls with no hydroxyapatite (10–34%) and osteoblasts cultured in the presence of hydroxyapatite (10–50%); however, D3+MK-7 was most effective.

**Figure 6:**
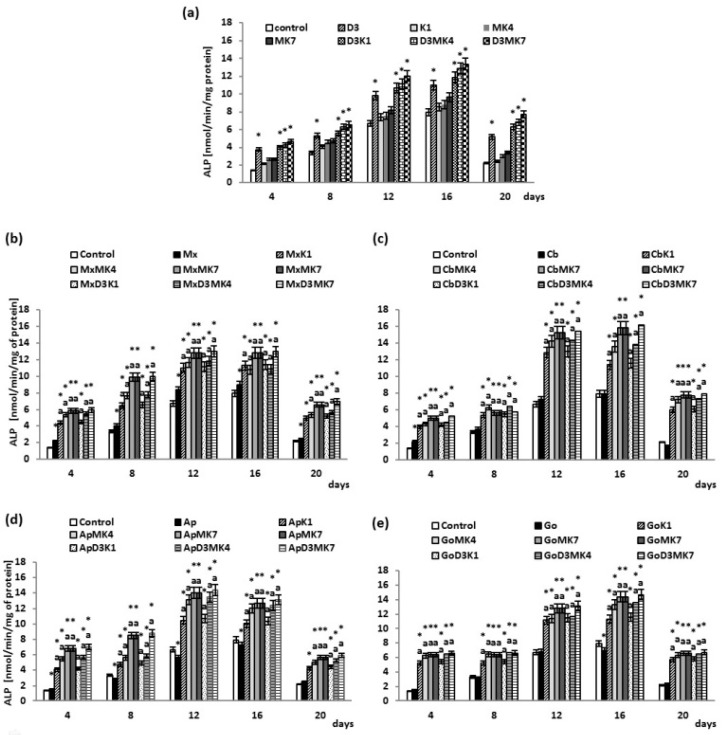
ALP activity in osteoblasts after incubation with hydroxyapatites and treated with vitamins D3, K1, MK4, and MK7 after 4, 8, 12, 16, and 20 days. The results are expressed as the nmol/min/mg of protein and are shown as the mean ± SD (*n* = 5). The values for the control cells and the treated cells were significantly different according to unpaired Student’s *t*-test. * Statistically significant differences versus control, *p* < 0.05; (**a**) statistically significant differences versus hydroxyapatites (Mx (Maxgraft), Cb (Cerabone), Ap (Apatos), Go (Gen-Os), respectively for graphs (**b**–**e**)), *p* < 0.05.

The osteoblast differentiation process, characterized by increasing ALP activity over time, reached a maximum after 16 days of culture, and thereafter, began to decrease (Figure 6). Vitamin D3 significantly enhanced ALP activity (40–150%) compared to the control (Figure 6a). Vitamin K1 and K2 did not affect ALP activity; however, the combination of vitamins D3+K1, D3+MK-4, and D3+MK-7 significantly increased ALP activity. At day 20, the largest increase was observed in MK-7-treated cultures, with a 2.5-fold increase. The presence of hydroxyapatite-based biomaterials did not significantly affect ALP activity, with the exception of an increase observed with Maxgraft (Figure 6b–e). Osteoblasts cultured with hydroxyapatite and treated with vitamin D3 exhibited significantly higher ALP activity compared to cells grown with only hydroxyapatite and control (no hydroxyapatite). Moreover, the addition of vitamin D3+K1, D3+MK-4 or D3+MK-7 led to a significant increase in ALP activity in the presence of hydroxyapatite. The combination of vitamin D3 and MK-7 led to the largest increase in ALP activity, particularly evident on day 4 (a two-fold to four-fold increase).

**Figure 7:**
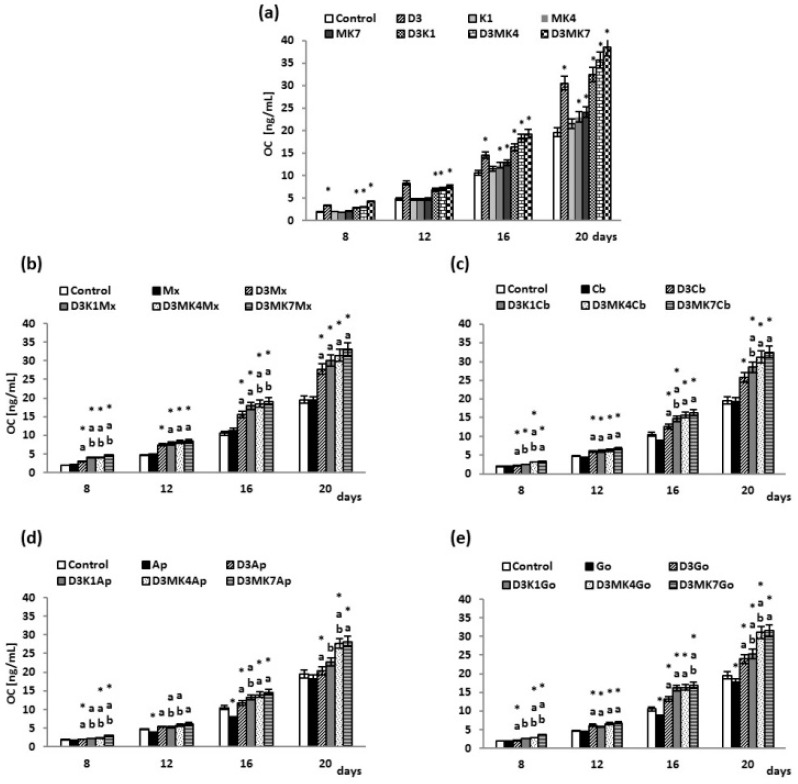
OC levels in osteoblasts after incubation with hydroxyapatites and treated with vitamins D3, K1, MK4, and MK7 after 8, 12, 16, and 20 days. The results are expressed as the ng/mL of medium and are shown as the mean ± SD (*n* = 5). The values for the control cells and the treated cells were significantly different according to unpaired Student’s *t*-test. * Statistically significant differences versus control, *p* < 0.05; (**a**) statistically significant differences versus group hydroxyapatites (Mx (Maxgraft), Cb (Cerabone), Ap (Apatos), Go (Gen-Os), respectively for graphs (**b**–**e**)), *p* < 0.05. (**b**) statistically significant differences versus group vitamin D_3_ with hydroxyapatites (Mx (Maxgraft), Cb (Cerabone), Ap (Apatos), Go (Gen-Os), respectively for graphs (**b**–**e**)), *p* < 0.05.

Cell culture media was collected, and OC levels were estimated as a marker of mineralization. The OC concentration gradually increased from day 8 to day 20 (Figure 7). Furthermore, synthesis of OC was enhanced by vitamin D3 treatment compared to control (Figure 7a). Vitamin K1 did not affect OC levels, whereas K2 caused an increase in OC production of 16–18% with MK-4 and 22–23% with MK-7, at day 16 and 20, respectively. The level of OC also increased in the cell culture media of D3+K1 (41–66%), D3+MK-4 (47–82%), and D3+MK-7 (55–116%) groups compared to controls. A decrease in OC concentration (approximately 10–26%) was observed in the media of osteoblasts cultures in the presence of Apatos and Gen-Os, resulting in similar OC levels (Figure 7b–e). Vitamin D3-treated osteoblasts cultured in the presence of hydroxyapatite had enhanced OC levels compared to controls and non-treated cells (hydroxyapatite only). Finally, exposing cells to D3+K1, D3+MK-4 or D3+MK-7 resulted in higher OC levels compared to controls and non-treated cells (hydroxyapatite only). The greatest enhancements were observed with vitamin D3 (55–120%) and MK-7 (40–120%).

## 4. Discussion

Hydroxyapatite-based biomaterials are often implanted to support bone regeneration or are used as a scaffold for culturing osteogenic cells, notably osteoblasts, which are essential for bone regeneration producing protein and mineral components of bone [50]. During the bone regeneration process, osteoblasts are recruited to the site of the bone defect, where they proliferate, differentiate, and contribute to extracellular matrix mineralization [51]. Biomaterials can cause inflammation and prolong wound healing as a result of increased ROS generation [52]. Vitamin D3, as well as K1, MK-4, and MK-7, possess osteoinductive, anti-inflammatory, and antioxidant properties, and may be able to prevent oxidative stress to improve bone regeneration [18,33,53].

Redox homeostasis results from the balance between ROS generation and the activity of cellular antioxidants [54]. Under physiological conditions, osteoblasts maintain redox homeostasis; however, interaction between osteoblasts and biomaterials can disturb this balance leading to oxidative stress. Results of this study showing that osteoblasts cultured in the presence of xenogeneic and allogeneic biomaterials exhibit increased ROS generation and reduced antioxidant capacity. Increased ROS production has been shown to directly affect the biological activity of osteoblasts via inhibition of Runx2 phosphorylation, the process responsible for transcription of type I collagen, osteopontin, osteoprotegin, sialoprotein, OC, and ALP [55]. 

Results of this study indicate that the presence of xenogeneic and allogeneic biomaterials can lead to antioxidant-related disorders in osteoblasts, including those associated with the GSH-dependent system. In addition to performing regulatory functions that ensure redox homeostasis, GSH tripeptide is also a cofactor of glutathione peroxidase, the main enzyme involved in breaking down peroxides including lipid peroxides. Thus, GSH is responsible for protecting cell membrane phospholipids and as a consequence, its deficiency can affect the function of biological membranes [56]. Lower GSH levels prevent glutathione peroxidase from performing its function; therefore, allogeneic biomaterials, in particular Apatos and Gen-Os, which lower GSH levels, can affect the activity of glutathione peroxidase, as observed in these studies. As a consequence, the GSH-dependent antioxidant system cannot properly perform its protective role and increased peroxidation of unsaturated fatty acids results in the formation of reactive aldehydes, such as 4-HNE, as confirmed by our study. Biomaterials based on bioactive glass have also been shown to alter the redox homeostasis in osteoblasts’ cells, manifested as increased 4-HNE production [57]. It should be mentioned that 4-HNE belongs to the α,β-unsaturated aldehydes, which include carbonyl groups and unsaturated bonds, thus, rendering them electrophilic and highly biologically reactive compounds mostly targeting nucleophilic compounds, such as GSH or proteins, leading to a reduction in biological activity including reduced GSH-Px phosphorylation [58,59]. This is also important because lipid peroxidation products decrease the expression of genes encoding for bone morphogenetic proteins (BMPs), including BMP2, BMP7, and BMP4, and therefore inhibit osteoblast maturation [55,60]. On the other hand, 4-HNE is known to act as growth regulating factor, acting in the concentration dependent and cell-type-specific manner influencing proliferation, differentiation, and apoptosis [61,62]. Mesenchymal cells, notably osteoblast-like cells, are especially sensitive to the growth regulating effects of 4-HNE, which interfere with complex bioactivities of numerous other growth-regulating factors (cytokines, hormones, etc.) and might eventually be involved in pathophysiology of altered bone growth, as in case of hypertrophic callus formation or otosclerosis [63,64]. Since 4-HNE was found to be produced by osteoblast-like cells, the growth of which was enhanced in vitro by bioactive glass, we may assume that production of 4-HNE by the osteoblasts observed in current study (also in the control cell cultures) supports previously described growth regulating effects of 4-HNE for the bone cells, while hydroxyapatite biomaterials resemble effects of bioactive glass [57,65]. However, to maintain functional growth of the bone cells it is necessary to enhance not only their proliferation but also differentiation, while oxidative stress and in particular 4-HNE were already proposed to be key mediators that might define the overall bioactivities of the implantable bioactive materials that require maintenance of the redox balance and the presence of supportive factors, like cytokines and vitamins [66,67]. 

Thus, the use of vitamins D3 and K to prevent redox imbalance could be beneficial for the bone growth and regeneration. Vitamin D3 did not have any effect on ROS level in this study, whereas ROS levels were significantly decreased with the addition of vitamin K to culture media, particularly the MK-7 homologue. The mechanism of vitamin K action may be associated with the inhibition of 12-LOX (lipoxygenase 12) activity [33], which can decrease ROS generation. Moreover, inhibiting ROS production may help maintain the metabolic activity of osteoblasts, but at the same time, could increase osteoclast activity, resulting in increased bone resorption [60]. This could reduce bone mineral density and the mechanical strength of newly formed and existing bone [68]. Therefore, vitamin K treatment can have associated consequences including oxidation of reduced glutathione to glutathione disulphide and reduced GSH-Px activity [69]. This is critical since GSH-Px activity protects membrane phospholipids and prevents the formation of 4-HNE. At the cellular level, oxidative damage disturbs cellular metabolism, thereby inhibiting osteoblast proliferation. This could be related to the oxidative damage to fibronectin, a glycoprotein that regulates certain osteoblast activities including adhesion, proliferation, migration, and cell morphology [70]. 

Here, we report decreased GSH levels in osteoblast cultures grown in the presence of allogeneic and xenogenic bone derivatives and treated with vitamin D3 and/or K. The decrease in GSH levels was particularly evident in cultures treated with the MK-7 homologue in combination with D3, which could be related to the vitamin D3-mediated increase in cysteine glutamate ligase (GCLC) activity which catalysis GSH biosynthesis [71]. Owing to the antioxidant properties of K vitamins, they can also affect GSH concentration, as confirmed by previous studies on nerve cells [34]. Higher GSH levels are accompanied by elevated GSH-Px activity, which enhances antioxidant activity in osteoblasts. Cells with enhanced antioxidant activity are less sensitive to oxidative damage and cell membrane damage. 

Our results suggest that vitamin D3 and vitamin K protect osteoblasts from oxidative stress and have beneficial effects on proliferation, differentiation, and mineralization of osteoblasts cultured both with and without hydroxyapatite-based biomaterials. This study suggests that vitamin K enhances the proliferative activity of osteoblasts (measured by DNA concentration). The MK-7 homologue is the most effective, either alone or combined with vitamin D3. Enhanced proliferation of MK-7-treated osteoblasts has been confirmed by other authors [72,73]. However, in previous studies, osteoblasts were not cultured in the presence of hydroxyapatite-based biomaterials. Osteoblast DNA levels have been associated with OC expression, osteoprotegerin, NFκB ligand (RANKL), and RANK ligand promoter genes facilitated by vitamin K [74,75]. Furthermore, the vitamin K family participates in sphingolipids synthesis, in addition to their structural role, sphingolipids take part in proliferation, differentiation, and intercellular recognition activities of neurons [76]. Vitamin D3 cannot affect proliferation on its own, but does support osteoblast differentiation, as demonstrated by increased expression of typical markers such as runt-related transcription factor 2 (RUNX2), type I collagen, alkaline phosphatase, and OC [77,78,79]. Our results show that unlike vitamin D3, the K vitamins do not influence osteoblast differentiation. However, the combination of vitamin K and D3 can significantly enhance osteoblast differentiation with increasing efficiency: K1 < MK-4 < MK-7. This can clearly be observed when osteoblasts are cultured in the presence of xenogeneic biomaterials (Maxgraft) and was previously observed by others [79]. Results of this study confirm previous reports of the participation of vitamin D3 and K vitamins in the bone regeneration process [75]. Human bone marrow stromal cells (hBMSC) and 2T3 osteoblast lines treated with vitamin D3 showed a concentration-dependent increase in ALP activity [80], whereas vitamin K did not affect ALP activity [42].

Osteocalcin is responsible for depositing calcium in bone tissue [81] and both vitamin D3 and vitamin K participate in the OC biosynthesis and activation processes [82]. In our study, vitamin D3 was shown to significantly affect OC levels in osteoblasts cultured without hydroxyapatite. A significant increase in OC synthesis was observed on day 12, indicating the start of the mineralization process. Other studies have confirmed the influence of vitamin D3 on OC production by osteoblasts [80]. However, we have shown that the presence of biomaterials, in particular, allogeneic bone-derived biomaterials (Apatos and Gen-Os), significantly decreases OC levels in the medium of osteoblast cultures. Furthermore, hydroxyapatite has been shown to bind 60–90% of OC [83]. Therefore, observed differences in the effects of different biomaterials on OC levels may be the result of varying levels of absorption related to the porosity and composition of the material. The lowest OC levels observed for osteoblasts cultured in the presence of Gen-Os is most likely owing to the presence of collagen in the biomaterial structure. Collagen content favours the absorption of OC since OC binds to the surface of collagen and further mediates the binding and accumulation of hydroxyapatite crystals on collagen fibers [40].

The K vitamin family supports biosynthesis of OC by participating in post-translational carboxylation of Gla residues of OC propeptides [26]. In contrast, carboxylated OC binds calcium to bone hydroxyapatite, thus supporting bone metabolism [81]. The results of our study indicate the effects of K vitamins are less significant than those of vitamin D3; however, the combination results in synergistic effects, even in the presence of allogeneic biomaterials (Apatos and Gen-Os). This could be because post-translational modification of OC depends on both vitamin D3 and vitamin K [37]. Vitamin D3 activates γ-glutamylcarboxylase for which vitamin K is a cofactor [26,84]. Moreover, vitamin K intensifies the synthesis and accumulation of carboxylated OC [82], thereby supporting hydroxyapatite deposition on collagen fibers. This process is regulated by the carboxylated form of OC [85]. Since vitamins D3 and K have a high affinity for hydroxyapatite, they are absorbed onto its surface and remain available to cells for a long time, despite the relatively short half-life of both vitamins [79]. Our results show intensified biosynthesis of OC between day 16 and 20 of the culture. Clinical studies have also confirmed that vitamin K (MK-4) and vitamin D3 treatments increase bone mass [86].

## 5. Conclusions

This study suggests that proliferation and differentiation of human osteoblasts in vitro was strongly influenced by redox balance, which might be altered in the presence of hydroxyapatite-based biomaterials. While physiological ROS production-induced lipid peroxidation generating 4-HNE might be important for the redox homeostasis of the growing osteoblasts, vitamins D3 and/or K could prevent their excessive production through oxidative stress of the osteoblasts induced by the biomaterials, thereby preventing their adverse effects on cellular proliferation and osteoblast differentiation. The most desirable effects on the maintenance of redox homeostasis were in our in vitro study observed for vitamins D3 and K2, especially the homologue MK-7. 

It is suggested that proliferation and differentiation of human osteoblasts in vitro is strongly influenced by redox balance, which is altered in the presence of hydroxyapatite-based biomaterials. ROS production and thus induced lipid peroxidation with 4-HNE generation might be important for the redox homeostasis of the growing osteoblasts, whereas vitamins D3 and/or K could prevent oxidative stress of the osteoblasts induced by the biomaterials, thereby preventing their adverse effects on proliferation and osteoblasts differentiation. The most desirable effects were in our in vitro study observed for vitamins D3 and K2, especially the MK-7 homologue.
